# Intelligent Industrial Process Control Systems

**DOI:** 10.3390/s23156838

**Published:** 2023-08-01

**Authors:** Iwona Grobelna

**Affiliations:** Institute of Automatic Control, Electronics and Electrical Engineering, University of Zielona Góra, 65-516 Zielona Góra, Poland; i.grobelna@iee.uz.zgora.pl

## 1. Introduction

The widespread realization of Industry 4.0 forces continuous progress in all its embedded technologies. Intelligent manufacturing systems are modern systems of manufacturing that integrate the abilities of humans, machines, and processes to achieve the best possible outcome. In this context, control processes directly influence the behavior of industrial systems. They are supposed to operate in a safe, reliable, and precise way. In order to ensure this, several modern technologies are combined together in an integrated design, involving artificial intelligence.

This Special Issue, belonging to the journal section “Industrial Sensors”, was dedicated to the newest interdisciplinary research in the area of intelligent industrial process control systems. A total of eight excellent research articles have been accepted and published, following a rigorous peer review process.

## 2. Summary of the Special Issue

The first paper [1] proposes a novel approach for the model checking of autonomous components within electric power systems specified by interpreted Petri nets. A formal specification enables the checking of some basic properties of the models, such as determinism or deadlock freedom, but also some behavioral user-defined properties. The requirements are written as temporal logic formulas and a rule-based logical model is used to support the verification process. The initial specification can then be formally verified, and any design errors can be identified at an early stage of electric power system development.

The second paper [2] introduced a data analysis and modelization method for the rolling mill process of manufacturing billets in steel plants. Based on a case study, two main problems were addressed: the data analysis of temperature sensors and current. The performed data analysis suggested necessary hardware modifications. The modelization phase provided the basis for future control and diagnosis applications that will exploit a temperature decay model.

The third paper [3] aims to improve actuator wear using noise filtering. It evaluates and measures the impact of noise filtering on the loop performance and on the actuator weariness. Relationships between the noise filtering time constant, loop performance, and valve travel deliver recommendations for control engineers. Suggestions for filter design are given, showing how far an engineer can go with filtering without a heavy loss of loop performance.

The fourth paper [4] exploits a decentralized PI/PID controller based on frequency domain analysis for two input–two output coupled tank systems. The fundamentals of the gain margin and phase margin were used to design the proposed controller. The robustness of the controller was verified by considering multiplicative input and output uncertainties. According to the authors, the proposed control algorithm exhibits better servo and regulatory responses compared to other existing techniques.

The fifth paper [5] presents a robust nonlinear current mode control approach for a pulse-width modulated DC-DC Cuk converter in a simple analog form. The control scheme is developed based on the reduced-state sliding-mode current control technique. The proposed controller does not require an output capacitor current sensor and double proportional–integral compensators as in conventional sliding-mode current controllers. Therefore, the cost and complexity of the practical implementation is minimized without degrading the control performance.

The sixth paper [6] proposes an optimization model of production change in a nonlinear supply chain system in emergencies. A three-level single-chain nonlinear supply chain system containing producers, distributors and retailers is established. The adaptive improved sliding mode controller is designed and used to construct an optimization model of the supply chain system under unexpected events. The effectiveness of the proposed method is verified using numerical simulation experiments.

The seventh paper [7] introduces an approach to minimize the maximum makespan of the integrated scheduling problem in flexible job shop environments, taking into account conflict-free routing problems. A hybrid genetic algorithm is developed for production scheduling and the optimal ranges of crossover and mutation probabilities are discussed. A case study of a real flexible job shop is used to present empirical evidence for the feasibility of the proposed approach.

The eighth paper [8] assesses industrial communication protocols to bridge the gap between machine tools and software monitoring. It presents an empirical study of three protocols: OPC-UA, Modbus, and Ethernet/IP. It aims to answer research questions about how these protocols differ in terms of performance and complexity of use from a software perspective, and how can their effectiveness be empirically evaluated and compared. 
